# Virus-like particle-based delivery of Cas9/guide RNA ribonucleoprotein efficiently edits the brachyury gene and inhibits chordoma growth in vivo

**DOI:** 10.1007/s12672-023-00680-9

**Published:** 2023-05-18

**Authors:** Yunping Hu, Baisong Lu, Zhiyong Deng, Fei Xing, Wesley Hsu

**Affiliations:** 1 Department of Neurological Surgery, Medical Center Boulevard, Wake Forest University School of Medicine, Winston-Salem, NC 27157 USA; 2 Medical Center Boulevard, Wake Forest University Institute for Regenerative Medicine, Winston-Salem, NC 27157 USA; 3 Department of Physiology and Pharmacology, Medical Center Boulevard, Wake Forest University School of Medicine, Winston-Salem, NC 27157 USA; 4 Department of Cancer Biology, Medical Center Boulevard, Wake Forest University School of Medicine, Winston- Salem, NC 27157 USA

**Keywords:** Brachyury, Chordoma, Genome editing, *In vivo* delivery, Virus-like particles

## Abstract

**Purpose:**

Chordoma is a rare and aggressive bone cancer driven by the developmental transcription factor brachyury. Efforts to target brachyury are hampered by the absence of ligand-accessible small-molecule binding pockets. Genome editing with CRISPR systems provides an unprecedented opportunity to modulate undruggable transcription factor targets. However, delivery of CRISPR remains a bottleneck for in vivo therapy development. The aim was to investigate the in vivo therapeutic efficiency of Cas9/guide RNA (gRNA) ribonucleoprotein (RNP) delivery through a novel virus-like particle (VLP) by fusing an aptamer-binding protein to the lentiviral nucleocapsid protein.

**Methods:**

The p24 based ELISA and transmission electron microscopy were used to determine the characterization of engineered VLP-packaged Cas9/gRNA RNP. The deletion efficiency of brachyury gene in chordoma cells and tissues was measured by genome cleavage detection assay. RT-PCR, Western blot, immunofluorescence staining, and IHC were employed to test the function of brachyury deletion. Cell growth and tumor volume were measured to evaluate the therapeutic efficiency of brachyury deletion by VLP-packaged Cas9/gRNA RNP.

**Results:**

Our “all-in-one” VLP-based Cas9/gRNA RNP system allows for transient expression of Cas9 in chordoma cells, but maintains efficient editing capacity leading to approximately 85% knockdown of brachyury with subsequent inhibition of chordoma cell proliferation and tumor progression. In addition, this VLP-packaged brachyury-targeting Cas9 RNP avoids systemic toxicities in vivo.

**Conclusion:**

Our preclinical studies demonstrate the potential of VLP-based Cas9/gRNA RNP gene therapy for the treatment of brachyury-dependent chordoma.

**Supplementary Information:**

The online version contains supplementary material available at 10.1007/s12672-023-00680-9.

## Introduction

Chordoma is a rare cancer that occurs in the skull-base, mobile spine, and sacrum. It often grows slowly but invasively. Surgical resection is the current standard of care, although the role of radiotherapy as a primary or adjuvant treatment continues to be explored [1]. After initial treatment, 50% of patients have local recurrence [2] and up to 40% develop distant metastases [3-5]. There is no first-line treatment approved for chordoma. Thus, there remains a significant unmet need for effective therapeutic modalities for chordoma.

Brachyury, a member of the T-box family of transcription factors, is a critical biomarker for chordoma. It plays an important role in early mesoderm formation and notochord development. Once the notochord regresses, brachyury expression is downregulated in most normal cell types [6]. High brachyury expression is found in chordoma cells [7-9] and plays a central role in cell viability and tumor-initiating capacity [10]. Brachyury is also associated with chemotherapy resistance, metastasis, and epithelial-to-mesenchymal transition [11, 12]. It may act as a key regulator of an oncogenic transcriptional network that mediates chordoma pathogenesis [13]. In addition, brachyury is rarely found in non-neoplastic adult tissue [6]. These studies suggest the potential of brachyury as a therapeutic target in chordoma.

Like other transcription factors, brachyury is difficult to be targeted pharmacologically, and a direct inhibitor of brachyury that may specifically control tumor cell fate has not been identified. A large-scale anti-chordoma drug screen identified molecules targeting brachyury function indirectly. For example, inhibitors targeting CDK [14, 15], EGFR/ERBB2 [16], KDM6A/KDM6B histone demethylases [17], and fibroblast growth factor receptor can downregulate brachyury expression [18]. However, strategies focused on indirect targeting of brachyury using small molecules have not demonstrated clinical efficacy in tumor repression thus far.

CRISPR)/ Cas9 genome editing can efficiently disrupt targeted genes but requires the effective delivery of genome editing tools into target cells. The ribonucleoprotein (RNP) complexes consisting of Cas9 protein and synthetic guide RNA (gRNA) has been used as a powerful method for delivery of CRISPR/Cas9 due to its fast editing kinetics, increased efficiency, and enhanced selectivity [19]. To date, electroporation remains the predominant approach used for delivering Cas9 RNPs into the intracellular environment, which may cause the loss of cell viability and an untargeted change in gene expression [20, 21]. Our team has recently developed a novel virus-like particle (VLP)-based system by fusing an aptamer-binding protein to the viral nucleocapsid protein to efficiently package Cas9/gRNA RNP [22, 23]. This innovative biotechnology combines the transient expression features of nanoparticle-delivery strategies that minimize off-target genomic alterations while retaining the transduction efficiency of lentiviral vectors. Here, we tested whether our developed VLP-based Cas9/gRNA RNP system could be used as a unique tool for direct gene editing of brachyury to treat chordoma. Our data show that the generated VLP-based system promotes the delivery of pre-formed Cas9/gRNA RNPs to chordoma cells and causes the transient expression of Cas9. The VLP-packaged brachyury-targeting Cas9 RNP demonstrates efficient gene editing leading to downregulation of brachyury expression and inhibition of chordoma growth in vivo with no evidence of systemic toxicity. These findings highlight that gene editing of brachyury with a VLP-packaged Cas9/gRNA conjugate may represent an attractive approach to treat chordoma.

## Materials and methods

### Reagents and antibodies

Cell culture media including IMDM (Cat# 12,440,053), DMEM (Cat# 11,966,025), DMEM/F12 (Cat# 113,200,330), and Opti-MEM (Cat# 31,985,070), One Shot™ Stbl3™ Chemically Competent E. coli (Cat# C737303), Lipofectamine 2000 (Cat# 11,668,027) and PureLink™ Genomic DNA Mini Kit (Cat# K18200) were purchased from Thermo Fisher Scientific (Grand Island, NY, USA). BbsI (Cat# R3539), BsaI (Cat# R3733), T4 Polynucleotide Kinase (Cat# M0201S), T4 Ligase (Cat# M0202S), and Taq DNA Polymerase (Cat# M0273L) were purchased from New England Biolabs (Ipswich, MA, USA). QIAquick Gel Extraction Kit (Cat# 28,704) and QIAGEN® Plasmid Mini Kit (Cat# 12,123) were purchased from QIAGEN (Germantown, MD, USA). The p24 based ELISA was purchased from Cell Biolabs (San Diego, California, USA). The CellTiter 96® AQueous One solution (Cat# G3582) was purchased from Promega (Madison, WI, USA). The protease inhibitor cocktail (Complete™, EDTA-free, Cat# 04693132001), phosphatase inhibitor cocktail (PhosStop, Cat# 4,906,845,001), ECL™ Prime Western Blotting Detection Reagent (Cat# GERPN2236), and Corning® Matrigel® Basement Membrane Matrix were purchased from Sigma-Aldrich (St. Louis, MO, USA). The Vectastain Elite ABC kit (Cat# PK-4001) and the ImmPACT® NovaRED® peroxidase substrate kit (Cat# SK-4805) were purchased from Vector Laboratories (Burlingame, CA, USA). The anti-Ki67 antibody (Cat# 2586) and Fluor 555-conjugated goat anti-mouse IgG (Cat# 4409 S) were purchased from Cell Signaling Technology (Danvers, MA, USA). The anti-brachyury (Cat# sc-166,962) and anti-Cas9 (Cat# SC-517,386) antibodies were purchased from Santa Cruz Biotechnology (Dallas, TX, USA).

### The design of gRNA and generation of lentiviral constructs

We designed the gRNAs using the online Benchling CRISPR gRNA Design tool (http://www.benchling.com). The gRNAs chosen were based on a high on-target score and a low off-target score. The parental plasmids pspCas9-3’UTR-ST2-com and px601-Tetra-com expressing Cas9 and sgRNAs have been described [22, 23]. The sequences for gRNAs shown in Supplementary  Table S1 were ligated into pspCas9-3’UTR-ST2-com and px601-Tetra-com by BbsI and BsaI restriction enzyme using T4 DNA ligase, respectively. Plasmids were transformed into competent cells of Stbl3 E. coli. The plasmid DNA was purified using QIAGEN® Plasmid Mini Kit. Positive clones were selected, and the sequences were verified using the primer 5′- GACTATCATATGCTTACCGT − 3′.

### Cas9/gRNA RNP VLP production

**Fig. 1:**
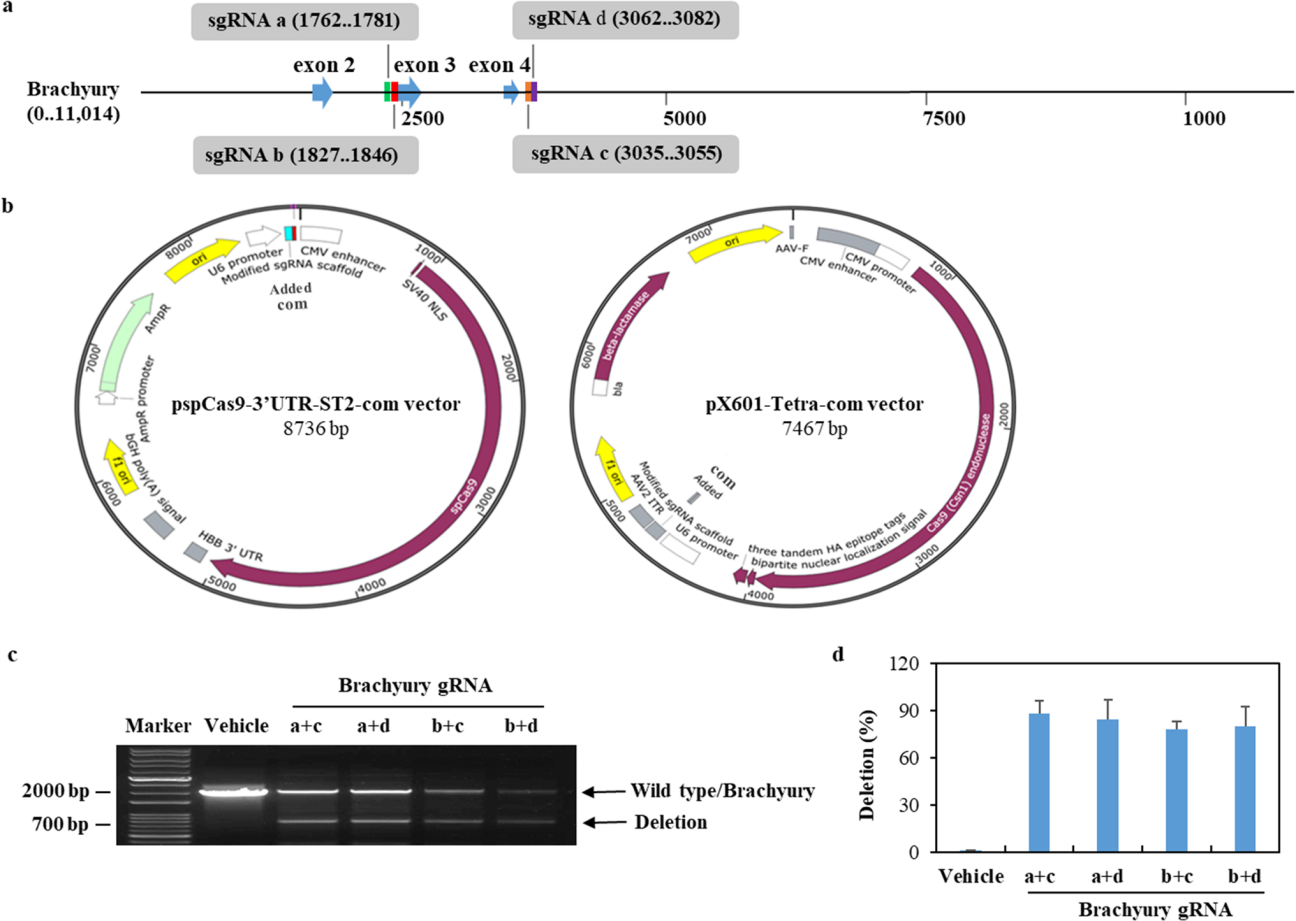
Modification of brachyury by Cas9-mediated brachyury sgRNA. **a** Targeting sites of the designed brachyury sgRNAs are colored as follows: (1) sgRNA a: green; (2) sgRNA b: red; (3) sgRNA c: orange, and (4) sgRNA d: purple. **b** The map of modified pspCas9-3'UTR-ST2-com vector and px601-Tetra-com vector expresses SpCas9 and sgRNA scaffold. **c** 293T cells were transfected with brachyury sgRNAs by Lipofectamine 2000. After 48 h, the genomic DNA from cells was isolated and amplified by PCR. The results were visualized by GelGreen® Nucleic Acid Gel Stain. **d** The efficiency of gene editing (mean ± SE, *n* = 3) was calculated by dividing the density of brachyury as total with the density of deletion

Cas9/gRNA RNP VLP was generated as described previously [22]. Briefly, 293T cells were seeded in a 10 cm dish with DMEM medium supplemented with 10% fetal bovine serum. After 24 h, the culture medium was replaced with 8 ml of Opti-MEM. A total of 7.5 µg of plasmids expressing brachyury targeting gRNAs a + c, b + c, a + d, or b + d (See Fig. 1A) were mixed with 7.5 µg of COM-modified packaging plasmid pspAX2-D64V-NC-COM and 7.5 µg of plasmid pMDLg (Addgene, Watertown, MA, USA) in 600 µl Opti-MEM. 40 µl of Fugene HD was then be added and incubated at room temperature for 10 min. The mixture was added into 293T cells. After 24-h transfection, the culture medium was changed with 15 ml of Opti-MEM. The supernatant containing Cas9 RNP VLP was collected after an additional 48-h transfection and spun for 5 min at 500 g to remove cell debris. The Cas9/gRNA RNP VLP in the supernatant was concentrated by ultracentrifugation at 100,000 g for 2 h at 4 °C. VLP pellets were resuspended in PBS. Cas9/gRNA RNP VLP was quantified by p24 based ELISA according to the manufacturer’s instructions.

### Chordoma cell culture and Cas9/gRNA RNP VLP transduction

Human chordoma cell lines JHC7 and UCH2 were purchased from the American Type Culture Collection (Manassas, VA, USA). JHC7 and UCH2 cells were seeded at 2 × 10^3^ cells/plate in 96-well plates, 2 × 10^4^ cells/plate in 24-well plates, or 2 × 10^5^ cells/plate in 6-well plates and cultured in DMEM/F12 and IMDM/RPMI 1640 (4:1) medium supplemented with 10% fetal bovine serum (FBS), respectively. After 24 h, cells were cultured with fresh warm culture medium and treated with 50 ng of Cas9/gRNA RNP VLP for an additional 48 or 72 h depending on the experiment.

### Transmission electron microscopy (TEM)

The suspension of Cas9/gRNA RNP VLP was added to carbon coated copper grids (200-mesh), and the particles were stained with phosphotungstic acid. The samples were dried and detected under TEM (Tecnai G2 30, FEI, Hillsboro, OR, USA).

### Genome cleavage detection assay

PureLink™ Genomic DNA Mini Kit was used to isolate the genomic DNA from cells treated with brachyury gRNAs or Cas9/gRNA RNP VLP. The specific cleavage site on DNA was generated by PCR amplification with specific primer sequences (Supplementary Table S2) that covers the Cas9 cut site. PCRs were performed on a 7500 Real-Time PCR System (Applied Biosystems) as described below: one cycle of 95 °C for 30 s, 30 cycles of 95 °C for 30 s, 57.5 °C for 1 min, 68 °C for 2 min, and one cycle of 68 °C for 5 min. Samples were analyzed by 1% agarose gel electrophoresis for cleavage, and deletion efficiencies were calculated by densitometry using ImageJ.

### Quantitative real-time PCR

Total RNA from chordoma cells treated with Cas9/gRNA RNP VLP was isolated using TRIZOL (Life Technologies Chemical, Ann Arbor, MI, USA) and then reverse transcribed using the Omniscript RT kit (Qiagen, Valencia, CA, USA), Oligo (dT) 12–18 Primer (Invitrogen, Carlsbad, CA), and RNase inhibitor (Promega, Madison, WI, USA). Amplification reactions were performed in triplicate in Applied Biosystems 7500 Real-Time PCR System using SYBR Green PCR Master Mix (Applied Biosystems, Foster City, CA, USA). Primers used are listed in Supplementary Table S2. Each assay included a standard curve of five serial dilutions to quantify gene expression. Data were normalized to GAPDH.

### Western blot assay

JHC7 and UCH2 cells (2 × 10^5^ cells/well) were seeded in 6-well plates for 24 h, then treated with Cas9/gRNA RNP VLP (50 ng) for up to 96 h. Aliquots of 20 µg of protein from cells were electrophoresed using 12.5% sodium dodecyl sulfate polyacrylamide gel electrophoresis and transferred to a nitrocellulose membrane. The blots were blocked with 5% non-fat dry milk at room temperature for 1 h and incubated overnight at 4 °C with the corresponding primary antibodies at dilutions recommended by the suppliers. Blots were subsequently incubated with horseradish peroxidase-conjugated secondary antibodies at room temperature for 1 h. The signal was detected using the ECL Prime western blotting detection reagent.

### Immunofluorescence staining

JHC7 and UCH2 cells (2 × 10^4^ cells/well) were plated in 24-well plates for 24 h, then treated with Cas9 RNP VLP (50 ng) for an additional 96 h. Cell were fixed with 10% formalin for 15 min and permeabilized with 0.2% Triton X-100 for 20 min. Primary antibodies (anti-Cas9 or anti-brachyury) were diluted 1:200 in permeabilization/blocking solution at 4 °C overnight. The secondary antibodies used were Alexa Fluor 555-conjugated goat anti-mouse IgG (1:1000). Cells were then stained with DAPI and observed using an immunofluorescence microscope.

### Cell growth assay

JHC7 and UCH2 cells were cultured in 96- or 6-well plates in triplicate at a density of 2 × 10^3^ cells or 2 × 10^5^ per well. After treatment with Cas9/gRNA RNP VLP (50 ng) for 48 h, cell growth was measured by MTS using a CellTiter 96® AQueous One solution based on the manufacturer’s protocol or by trypan blue exclusion assay as described previously [24]. Data represent the mean absorbance of three wells, and these are presented relative to control.

### In vivo mouse xenograft models

Six-week-old Crl:SHO-Prkdc^scid^Hr^hr^ SCID nude female mice were purchased from Charles River Laboratories (Ann Arbor, MI, USA). Mice were inoculated subcutaneously with 1 × 10^6^ UCH2 cells in 100 µl PBS mixed with 100 µl Matrigel into the right flank. Tumors were allowed to establish for 25 days (~ 150 mm^3^). Mice were randomly divided into three treatment groups (n = 10/group) and intratumorally injected with (1) VLP-packaged Cas9/control gRNA (primary pspCas9-3’UTR-ST2-com and px601-Tetra-com vectors, 1 mg/kg body weight) on days 25 and 28, (2) VLP-packaged Cas9/brachyury gRNAs a + c (1 mg/kg body weight) on days 25 and 28, and (3) VLP-packaged Cas9/brachyury gRNAs a + c (1 mg/kg body weight) on days 25, 28, 31, and 34. The overall volume injected during each intratumoral treatment was < 100 ul. Tumor size was measured by electronic caliper, and volume was calculated as volume = (length × width^2^)/2. Tumor growth and body weights of mice were measured every 3 days after initial treatment. Animals were euthanized on day 41 after injection of UCH2 cells. Blood, tumor tissues, and organs from mice were collected for further assays. All procedures were approved by the Institutional Animal Care and Use Committee of Wake Forest University School of Medicine (protocol A21-025).

### Histology and immunohistochemistry (IHC)

Tissue sections from in vivo mouse xenograft models were fixed in 10% buffered formalin and embedded in paraffin, then sectioned at 5 μm and mounted on positively charged glass slides. Sections from mice organs were stained with hematoxylin and eosin (H & E). The slides from tumor tissues were deparaffinized and exposed to heat-induced antigen retrieval in 10 mM sodium citrate buffer (pH 6.0) for 10 min. Each tumor tissue sample was stained with primary anti-brachyury or anti-Ki67 antibodies as well as a biotin-conjugated secondary antibody. Visualization of the measured protein was performed using the Vectastain Elite ABC kit. Positive cells for brachyury and Ki67 were quantified by ImageJ software.

### Blood biochemistry

Mouse blood was collected without using an anticoagulant and was allowed to clot for 2 h at room temperature. The serum was then separated by centrifugation at 2000 g for 15 min. The levels of alanine aminotransferase (ALT), aspartate aminotransferase (AST), and blood urea nitrogen (BUN) in serum from tumor-bearing mice were analyzed using the commercially available kits (Cat# MAK052-1KT, MAK055-1KT and MAK006-1KT, Sigma-Aldrich, St. Louis, MO, USA).

### Statistical analysis

Data are expressed as means ± SD of at least triplicate measurements or SEM of at least three independent experiments. Statistical analysis was performed by SPSS V.10.0 for Windows using one-way analysis of variance with Bonferroni post-hoc test. *P* < 0.05 was considered statistically significant.

## Results

### Cas9/brachyury gRNA efficiently targets brachyury

Previous studies have shown that the use of dual single gRNA (sgRNA) to target a gene can significantly improve the efficiency of Cas9-mediated gene editing [25]. Therefore, we selected 2 pairs of targeted sites in the brachyury gene (Fig. 1a). Oligos of sgRNA a and b were for SpCas9 and cloned into a pspCas9-3’UTR-ST2-com vector. The oligos of sgRNA c and d were for SaCas9 and cloned into a px601-Tetra-com vector. The sgRNA scaffolds in pspCas9-3’UTR-ST2-com and pX601-Tetra-com vectors were modified by inserting a com aptamer into their respective gRNA scaffold (Fig. 1b). To detect the efficiencies of sgRNAs targeting brachyury, we transfected our generated sgRNA expression plasmid DNA into 293T cells. The genomic DNA from 293T cells was used to detect the presence of site-specific cleavage by PCR. As shown in Fig. 1c and d, and Supplementary Fig S1, efficient deletion (78–88%) was found after transfection with the dual brachyury sgRNAs, demonstrating that the selected sgRNAs worked specifically and effectively with Cas9 to edit the brachyury gene.

### The VLP-based Cas9/gRNA RNP system allows for transient Cas9 expression in chordoma cells

**Fig. 2:**
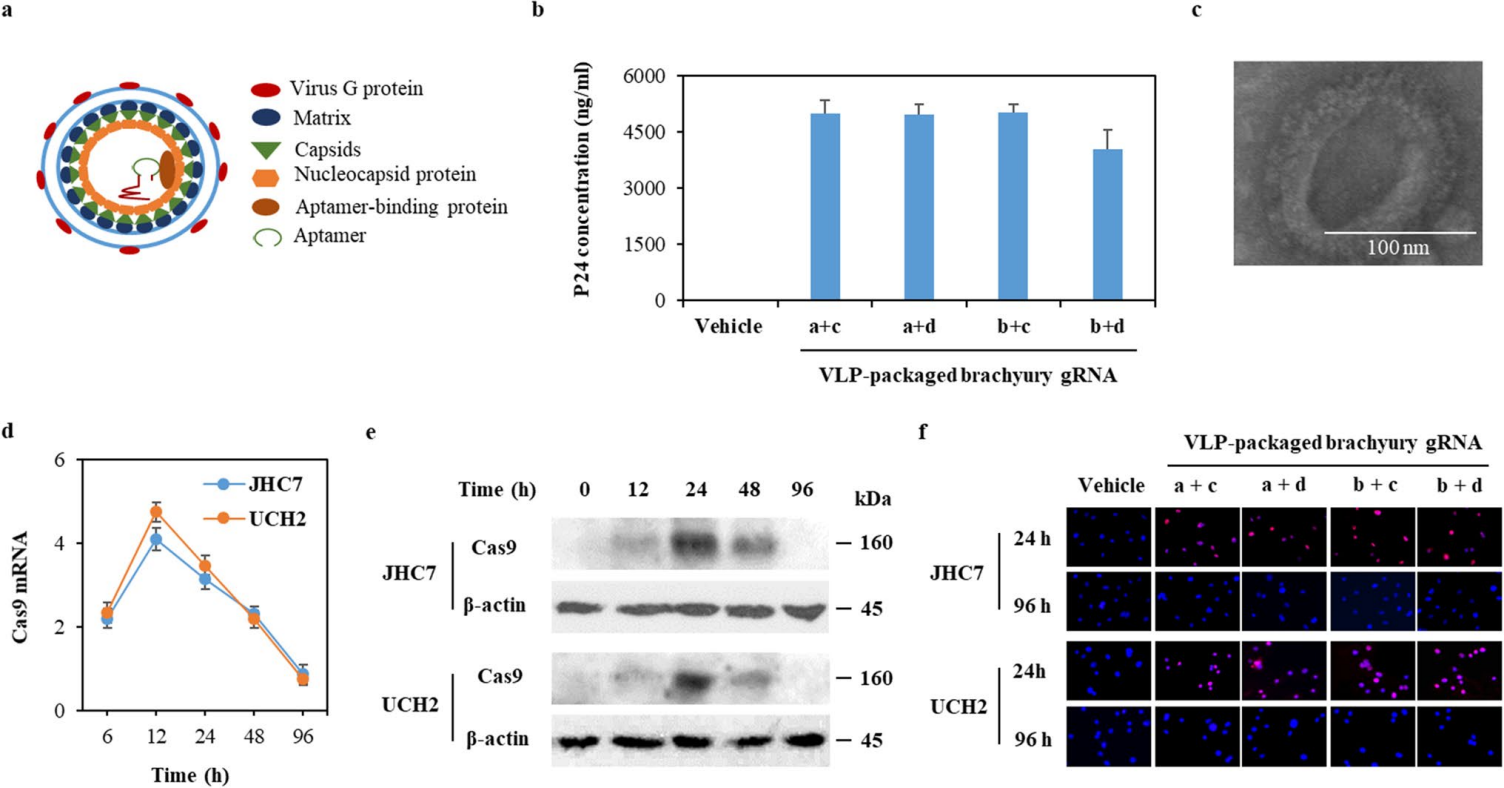
Characterization of VLP-packaged brachyury gRNA. **a** The schematic diagram of development of Cas9/gRNA RNP for gene editing of brachyury. Aptamer-binding proteins (ABP) are incorporated into VLP via fusing ABP with the lentiviral nucleocapsid protein (NC). The corresponding aptamer was added into sgRNA, which complexes with Cas9 protein and forms RNP during lentiviral cap assembly. The RNP was packaged into the lentiviral capsids (CA)/Matrix (MA) via the specific aptamer/ABP interaction. **b** 293-T cells were transfected with ABP-modified plasmids and our generated aptamer-modified gRNA expression vectors. The VLP-packaged Cas9/brachyury gRNA RNP in medium supernatant (mean ± SD, *n* = 3) was measured by calculating the lentivirus productions using p24 ELISA assay. **c** Representative transmission electron microscope image of VLP-packaged brachyury gRNA. **d** JHC7 and UCH2 cells (2×10^5^) were treated with vehicle (medium) or VLP-packaged brachyury gRNAs (50 ng) for the indicated time point. Total RNA and protein from cells were prepared. Cas9 gene expression (Mean ± SD, *n* = 3) was measured by qRT-PCR. **e** JHC7 and UCH2 cells were treated with VLP-packaged brachyury gRNA a+c for up to 96 h. Protein from cells were prepared. Cas9 expression was measured by Western blot. **f** Immunofluorescence staining of Cas9 (red) in JHC7 and UCH2 cells transduced with VLP-packaged Cas9/brachyury gRNAs for 24 h and 96 h. Nuclei were counter-stained with DAPI (blue)

To produce “all-in-one” Cas9/gRNA RNP VLPs (Fig. 2a), 293T cells were co-transfected with ABP-modified packaging plasmid pspAX2-D64V-NC-COM, plasmid pMD1g and our generated aptamer-modified gRNA expression vectors. After 48-h transfection, we found that Cas9/gRNA RNP was highly concentrated in supernatants (Fig. 2b). Transmission electron microscopy analysis found numerous membrane structures in our VLP-packaged Cas9/brachyury gRNA conjugates, confirming the characterization of membrane structures in VLP (Fig. 2c). The average diameter of VLP-packaged Cas9/brachyury gRNA preparations was ~ 120 nm. Our in vitro studies have also demonstrated the time course of Cas9 expression in chordoma cells transduced with VLP-packaged Cas9/gRNA. The level of Cas9 mRNA in chordoma cells was the highest at 12-h transduction, then decreased considerably at 96 h (Fig. 2d). The translation of mRNA takes time, so Cas9 protein expression was low at 12 h, increased significantly at 24 h, then decreased to an undetectable level at 96 h (Fig. 2e and f, and Supplementary Fig S2). These results demonstrate that our engineered VLP allows for rapid and transient delivery of Cas9/gRNA RNPs to chordoma cells.

### VLP-packaged brachyury-targeting Cas9 RNP demonstrates efficient gene editing in chordoma cells leading to downregulation of brachyury expression and inhibition of cell growth

**Fig. 3:**
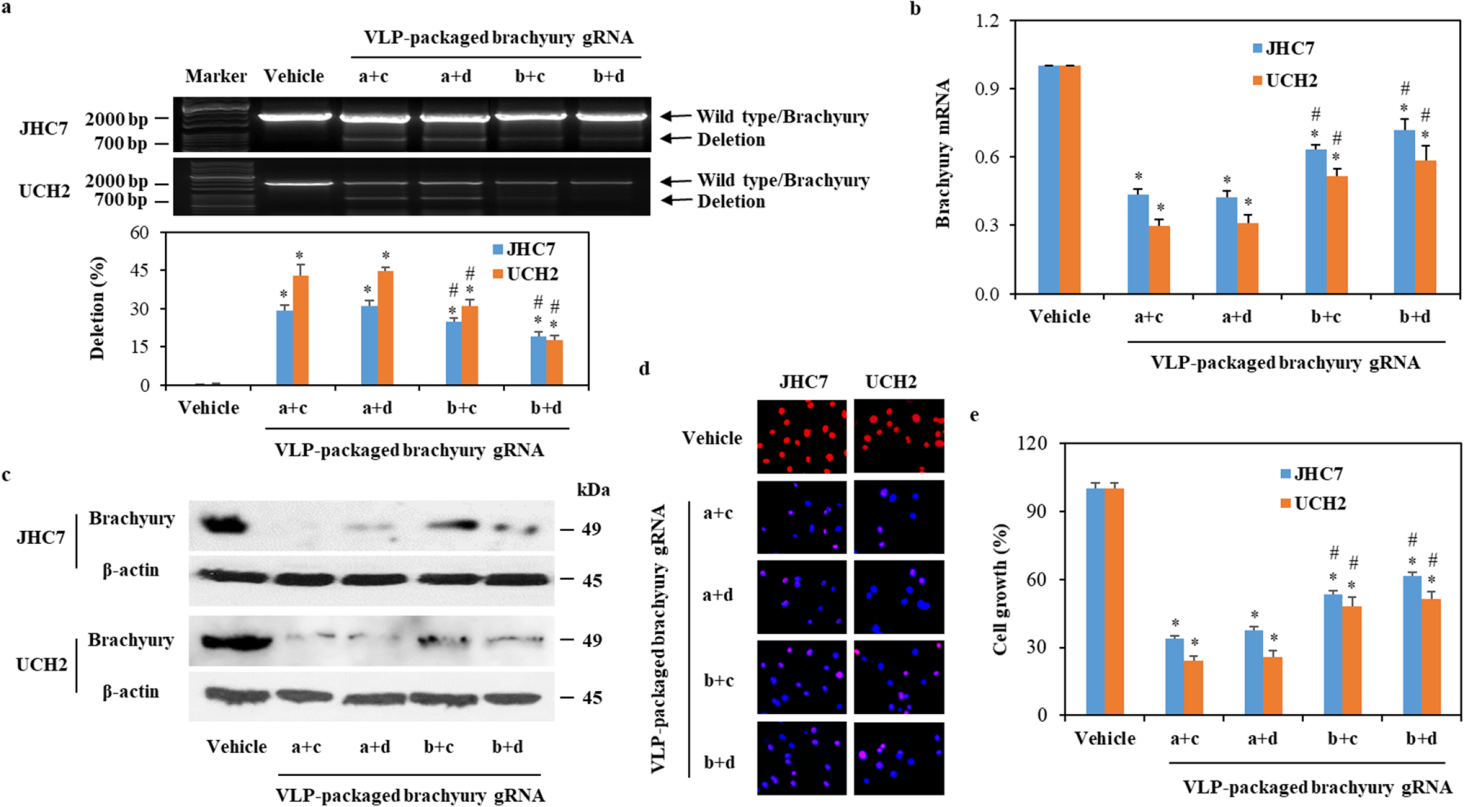
The efficiency of VLP-packaged brachyury gRNA in targeting the brachyury gene in chordoma cells. **a** The representative band of deletion in JHC7 and UCH2 cells treated with 50 ng of VLP-packaged brachyury gRNAs for 48 h. The efficiency of gene editing (mean ± SE, *n* = 3) was calculated by dividing the density of brachyury as total with the density of deletion. **b**, **c**, **d** and **e** JHC7 and UCH2 cells were treated with vehicle (medium) or VLP-packaged brachyury gRNAs (50 ng) for 48 h. Brachyury gene (**b**) and protein expressions (**c**) were measured by qRT-PCR and Western blot, respectively. Immunofluorescence staining was used to detect brachyury expression (red) (**d**). Nuclei were counter-stained with DAPI (blue) (**d**). Cell growth was measured by MTS assay (**e**). Data were presented as mean ± SD (*n* = 3). * *P* < 0.05, vs Vehicle in the same cell line; # *P* < 0.05, vs a+c or a+d in the same cell line

To test the efficiency of VLP-packaged brachyury-targeting Cas9 RNP in gene editing for chordoma cells, we cultured two human chordoma cell lines (JHC7 and UCH2) with VLP-packaged Cas9/brachyury gRNA conjugates. As shown in Fig. 3a and Supplementary Fig S3a, cleavage bands were observed in VLP-packaged Cas9/brachyury gRNA treatment groups but not in the control group. In addition, VLP-packaged Cas9/brachyury gRNA RNP a + c and a + d were more efficient at decreasing brachyury gene and protein expression (Fig. 3b, c and d, and Supplementary Fig S3b) and chordoma cell growth (Fig. 3e and Supplementary Fig S3c) compared to VLP-packaged Cas9/brachyury gRNA RNP b + c and b + d. Interestingly, protein expression (Fig. 3c and d) is significantly inhibited in all Cas9 RNPs despite variability in the efficacy of brachyury gene deletion (Fig. 3b). One explanation for this observation is that single cuts may also generate small insertions and deletions, which disrupts the reading frame and protein expression.

### Intratumoral injection of VLP-packaged brachyury-targeting Cas9 RNP downregulates brachyury expression and inhibits chordoma progressionin vivo

**Fig. 4:**
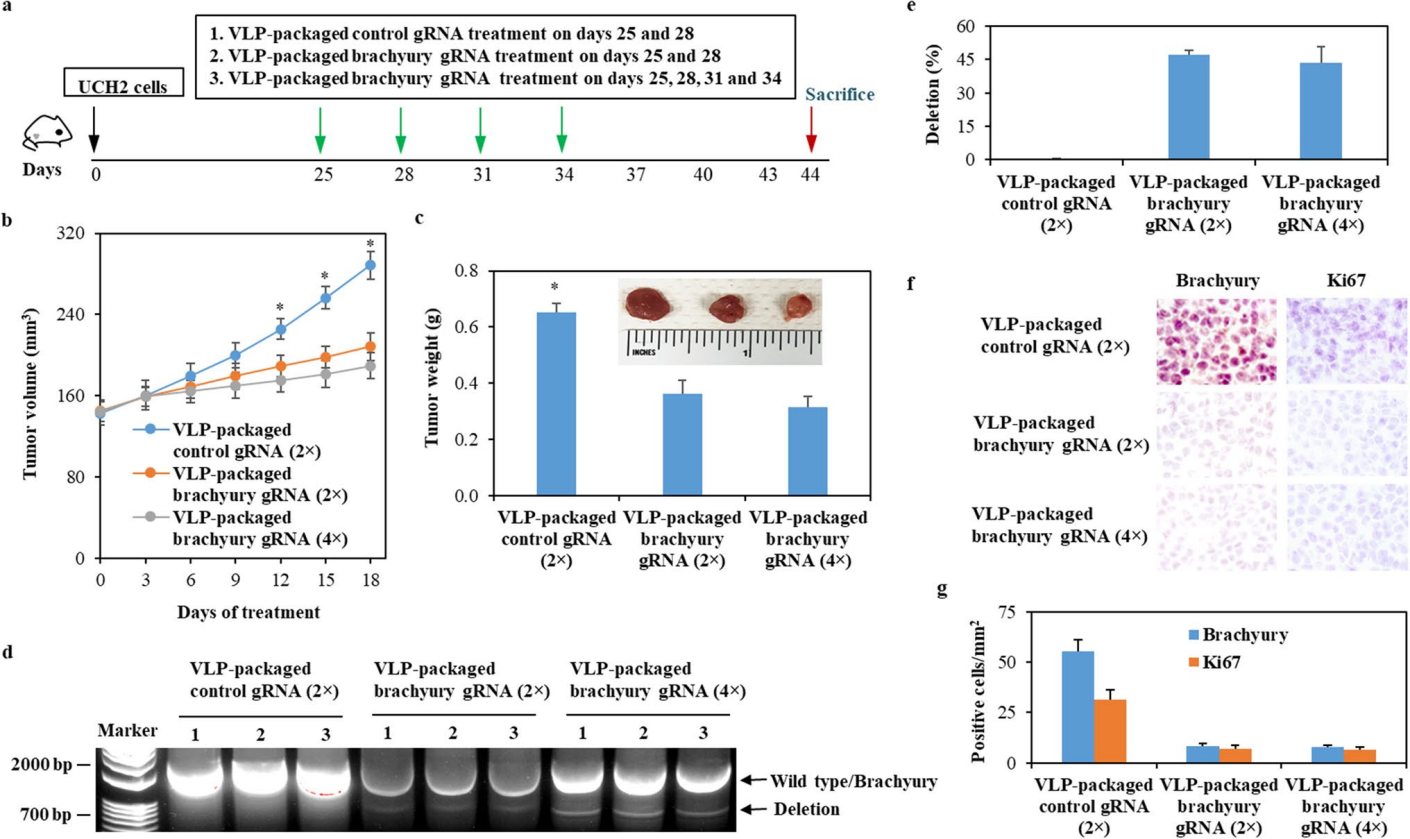
The therapeutic efficiency of VLP-packaged Cas9/brachyury gRNA in xenograft mouse model. **a** The schematic diagram of xenograft mouse model and treatment timeline. **b** and **c** UCH2 cells (2×10^6^) were subcutaneously injected into NOD SCID mice. Mice were intratumorally injected with VLP-packaged Cas9/control gRNA (1 mg/kg body weight) or VLP-packaged Cas9/brachyury gRNA a+c (1 mg/kg body weight) on 3-day intervals [a total of 2 (2×) or 4 times (4×)]. Tumor growth (mean ± SD, *n* = 10) was evaluated by measuring tumor volume (**b**) and tumor weight (**c**). * *P* < 0.05 vs VLP-packaged Cas9/brachyury gRNA. **d** The genomic DNA from tumor tissues was isolated and amplified by PCR. The representive genome cleavage visualized by GelGreen® Nucleic Acid Gel Stain was presented. **e** The efficiency of in vivo gene editing (mean ± SD, *n* = 10) was calculated by dividing the density of brachyury as total with the density of deletion. **f** and **g** Brachyury and Ki67 protein expression in tumor tissus were determined by IHC staining using the anti-brachyury and anti-Ki67 antibody (**f**) and expressed (mean ± SD, *n* = 10) as number of positive cells per mm^2^ of tumor (**g**)

Intratumoral chemotherapy for chordoma has been explored previously [26]. To investigate the in vivo efficiency of the delivery strategy using the VLP-packaged Cas9/brachyury gRNA, we generated a subcutaneous chordoma mouse model and directly injected VLP-packaged Cas9/ primary vectors as control or VLP-packaged Cas9/brachyury gRNA into tumors (Fig. 4a). We found that serial intratumoral injections of VLP-packaged Cas9/brachyury gRNA significantly inhibited tumor growth (Fig. 4b and c), specifically deleted the brachyury gene (Fig. 4d and e), and reduced expressions of brachyury and Ki67 (Fig. 4f g) compared to VLP-packaged primary vectors. These data suggest that in vivo delivery of brachyury-targeting Cas9 RNP by VLP has the potential to be a therapeutic option for controlling chordoma progression via specific genomic deletion of brachyury.

### VLP-packaged brachyury-targeting Cas9 RNP produces limited adverse effectsin vivo

**Fig. 5:**
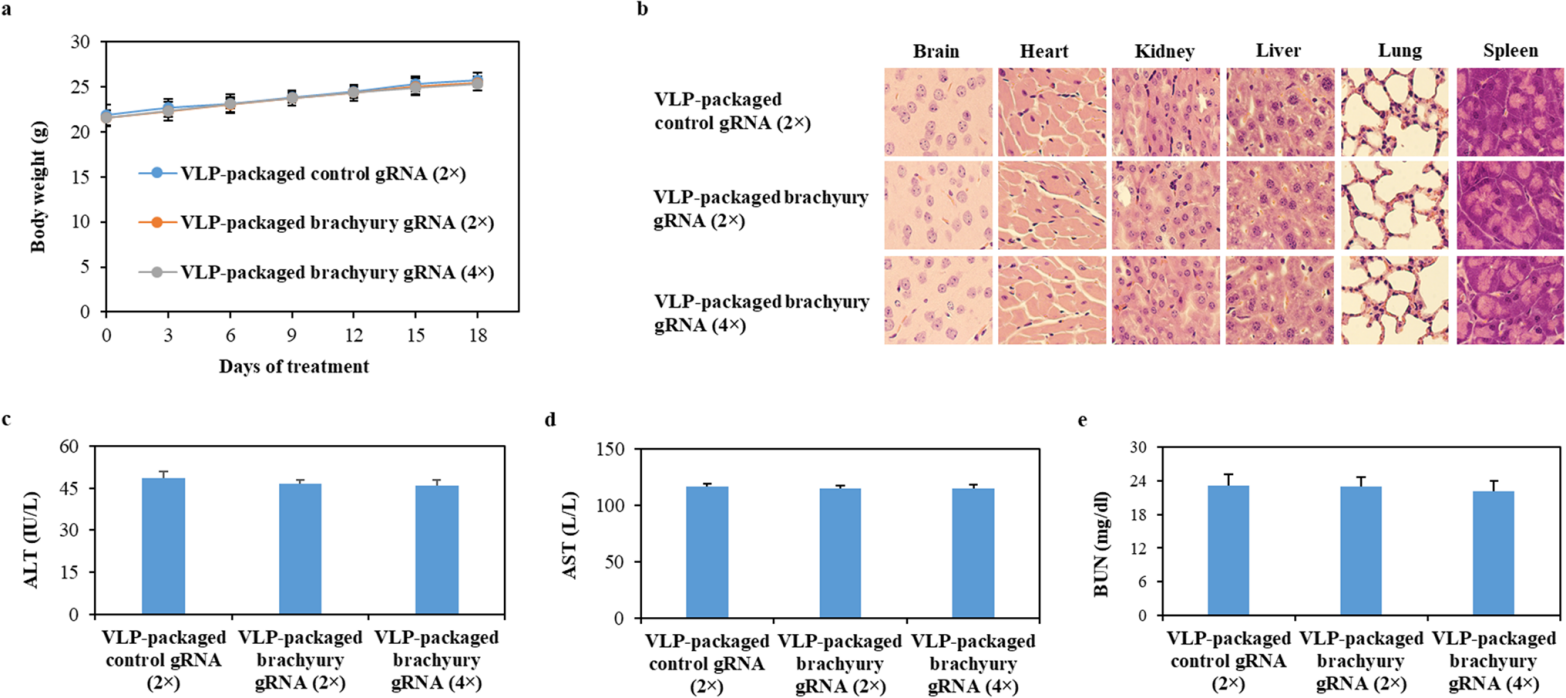
The detection of systemic toxicity of VLP-packaged brachyury gRNA in xenograft mouse model. **a** Body weights of mice were measured during the study period. **b** The representative H&E staining of brain, heart, kidney, liver, lung, and spleen sections from mice at the endpoint is shown. **c**, **d**, and **e** The levels of serum ALT, AST, and BUN (mean ± SD, *n* = 10) measured by colorimetric assay

To assess the potential toxicities of administered VLP-packaged brachyury-targeting Cas9 RNP, we monitored body weight of mice and performed histopathological analysis of brain, heart, kidney, liver, lung, and spleen of mice in each treatment group. We found no evidence of differences in body weight (Fig. 5a) and organ histology (Fig. 5b) due to VLP-packaged Cas9/brachyury gRNA treatment (Fig. 5b). Additionally, we found that all mice exhibited ALT, AST, and BUN levels within the normal ranges, and there were no discernible differences between VLP-packaged Cas9/primary vector-treated mice and VLP-packaged Cas9/brachyury gRNA-treated mice (Fig. 5c and d, and 5e). These results demonstrate that intratumoral administration of VLP-packaged Cas9/brachyury gRNA does not lead to significant systemic toxicity.

## Discussion

Brachyury is an ideal therapeutic target for the treatment of chordoma due to its driving role in chordoma development and lack of expression in normal adult tissues. Unfortunately, there are no treatment options available to target brachyury directly. The CRISPR/Cas9 genome editing technology can precisely manipulate cancer cell genomes to correct or eliminate cancer-causing aberrations, which may open new possibilities to develop brachyury-targeted options to limit chordoma progression. In this study, we show for the first time that administration of VLP transiently delivered Cas9/gRNA RNP to chordoma cells leading to efficient and specific brachyury gene deletion with subsequent inhibition of chordoma cell proliferation and tumor progression in xenograft models. In addition, the VLP-packaged brachyury-targeting Cas9 RNP did not induce systemic toxicities in vivo. Our study provides proof-of-concept that the VLP-based Cas9/brachyury gRNA RNP system can be utilized to efficiently target brachyury in chordoma.

The CRISPR/Cas9 system has been successfully applied to in vitro cancer research by inhibiting one or more oncogenic molecular pathways. However, the in vivo use of the CRISPR/Cas9 system as a therapeutic, particularly with respect to engineering optimal methods of delivery, is in its infancy [27]. Delivering an RNP is the most straightforward strategy [28], as there is no need for a transcription or translation process [29, 30]. It immediately initiates gene editing when entering the cell, significantly reducing off-target effects and immune responses [29]. Previous studies have shown that VLPs as a delivery vehicle for RNP cargoes exploit the efficiency and tissue targeting advantages of viral delivery [22, 31, 32]. However, existing VLP-mediated strategies for delivering gene editing agent RNPs support only modest editing efficiencies with limited validation of therapeutic efficacy in vivo [33]. In this study, we engineered a new VLP by fusing ABP with the lentiviral nucleocapsid protein (NC) within the group-specific antigen (Gag) that can efficiently infect cells and inserting RNA aptamer into the gRNA scaffold. Our study shows that our engineered VLP efficiently delivers Cas9/gRNA RNP components into chordoma cells and tissues and achieves therapeutic levels of brachyury gene editing leading to significant inhibition of chordoma growth in vivo. These results suggest that our VLP is a promising vehicle for in vivo Cas9/gRNA RNP-based gene editing to treat chordoma.

Local administration of CRISPR/Cas9 therapeutics has shown promising therapeutic outcomes [34-36]. We therefore intratumorally delivered Cas9/gRNA RNP via VLP to treat chordoma using mouse xenograft models. Our data demonstrates that serial intratumoral administration causes brachyury deletion leading to approximately 85% knockdown of brachyury in vivo (Fig. 4g) and significant tumor growth inhibition. Interestingly, the dose-effect relationship is not significant, which may be explained by the short time of observation or a maximum effect achieved by a low dose (2-time administration). Our in vivo findings suggest that intratumorally delivered gene editing for brachyury with VLP-based Cas9/gRNA RNP could be a valid treatment strategy for chordoma.

Safe delivery of CRISPR/Cas9 components is a prerequisite for successful gene editing therapies. Cytotoxic side effects and/or off-target events have been described for overexpression or long-term expression of DNA-modifying enzymes [37, 38]. Therefore, the CRISPR/Cas9 complex should ideally be transiently expressed to avoid stable and long-term expression of this DNA-modifying enzyme. The need for transient designer nuclease expression in gene editing has promoted attempts using VLPs to deliver both in vitro and in vivo therapeutic RNPs. In this study, we assembled a novel VLPs-based Cas9/gRNA RNP system, which can obtain 90 ~ 100% capsid assembly efficiency of normal viral vectors and result in over 80% INDEL rates and < 1% INDELs rates on an off-target with 1 nucleotide mismatch [22, 39]. Here, we further addressed the safety concerns in a situation where a maximum editing efficiency is achieved in the context of an in vivo chordoma model. Our in vitro and in vivo studies demonstrate that the VLP-based Cas9/gRNA RNP system allows for short-term (96 h) expression of Cas9 in chordoma cells while maintaining gene editing capacity and avoiding systemic toxicities, suggesting that our VLP-delivered Cas9/gRNA RNP-based gene editing therapy for brachyury-dependent chordoma is safe and effective.

Chordoma is currently classified by The World Health Organization (WHO) into three subtypes with variable and distinctive morphology: conventional/chondroid, poorly differentiated and dedifferentiated chordoma [40]. The predominant subtype of chordoma (accounting for approximately 75% of cases) is conventional chordoma. Multiple studies have shown the importance of brachyury in the pathogenesis of chordoma [14, 15, 41]. However, some poorly differentiated and dedifferentiated chordoma display the loss of brachyury [42, 43], SMARCB1/INI1 [44, 45] or H3K27 trimethylation [46], TP53 mutation [42], and NF-ĸB activation [43]. These data suggest that the most common subtype of chordoma may potentially benefit from our VLP-delivered Cas9/gRNA RNP-based gene editing therapeutic approach, whereas poorly differentiated and dedifferentiated chordoma would not benefit from this approach.

Although brachyury gene editing did not lead to tumor regression, it is possible that slowing chordoma growth may in and of itself be sufficient to improve patient outcomes. This may be particularly true in situations where chordoma is located near critical neurological structures not amenable to safe surgical resection or optimal radiotherapy. Patients with recurrent chordoma that are not amenable to further surgery or radiation may potentially benefit from strategies that slow tumor growth. It is also possible that brachyury gene editing may be a valuable adjuvant therapy in combination with chemo/immunotherapy or radiation, which are important questions under active investigation.

## Conclusion

This study established that our packaged VLP-based Cas9/gRNA RNP enabled efficient in vitro and in vivo editing of the brachyury gene in chordoma. In addition, we showed that VLPs offer transient and safe delivery of Cas9/gRNA RNP to chordoma cells and tissues to inhibit tumor progression. Our data provide important preclinical evidence of the efficacy and safety of the VLP-based Cas9/gRNA RNP therapeutic approach, implying that gene editing of brachyury may provide a novel therapeutic paradigm for chordoma patients that warrants further investigation.

## Supplementary Information

Additional file  1 

## Data Availability

Data are available upon reasonable request.

## References

[1] Tobert DG, Kelly SP, Xiong GX, Chen YL, MacDonald SM, Bongers ME (2022). The impact of radiotherapy on survival after surgical resection of chordoma with minimum five year follow-up. Spine J.

[2] Stacchiotti S, Gronchi A, Fossati P, Akiyama T, Alapetite C, Baumann M (2017). Best practices for the management of local-regional recurrent chordoma: a position paper by the Chordoma Global Consensus Group. Ann Oncol.

[3] Bergh P, Kindblom LG, Gunterberg B, Remotti F, Ryd W, Meis-Kindblom JM (2000). Prognostic factors in chordoma of the sacrum and mobile spine: a study of 39 patients. Cancer.

[4] Volpe R, Mazabraud A (1983). A clinicopathologic review of 25 cases of chordoma (a pleomorphic and metastasizing neoplasm). Am J Surg Pathol.

[5] Gay E, Sekhar LN, Rubinstein E, Wright DC, Sen C, Janecka IP (1995). Chordomas and chondrosarcomas of the cranial base: results and follow-up of 60 patients. Neurosurgery..

[6] Palena C, Polev DE, Tsang KY, Fernando RI, Litzinger M, Krukovskaya LL (2007). The human T-box mesodermal transcription factor brachyury is a candidate target for T-cell-mediated cancer immunotherapy. Clin Cancer Res.

[7] Vujovic S, Henderson S, Presneau N, Odell E, Jacques TS, Tirabosco R (2006). Brachyury, a crucial regulator of notochordal development, is a novel biomarker for chordomas. J Pathol.

[8] Scheil-Bertram S, Kappler R, von Baer A, Hartwig E, Sarkar M, Serra M (2014). Molecular profiling of chordoma. Int J Oncol.

[9] Oakley GJ, Fuhrer K, Seethala RR, Brachyury (2008). SOX-9, and podoplanin, new markers in the skull base chordoma vs chondrosarcoma differential: a tissue microarray-based comparative analysis. Mod Pathol.

[10] Shah SR, David JM, Tippens ND, Mohyeldin A, Martinez-Gutierrez JC, Ganaha S (2017). Brachyury-YAP Regulatory Axis drives stemness and growth in Cancer. Cell Rep.

[11] Huang B, Cohen JR, Fernando RI, Hamilton DH, Litzinger MT, Hodge JW (2013). The embryonic transcription factor brachyury blocks cell cycle progression and mediates tumor resistance to conventional antitumor therapies. Cell Death Dis.

[12] Fernando RI, Litzinger M, Trono P, Hamilton DH, Schlom J, Palena C (2010). The T-box transcription factor brachyury promotes epithelial-mesenchymal transition in human tumor cells. J Clin Invest.

[13] Nelson AC, Pillay N, Henderson S, Presneau N, Tirabosco R, Halai D (2012). An integrated functional genomics approach identifies the regulatory network directed by brachyury (T) in chordoma. J Pathol.

[14] Sharifnia T, Wawer MJ, Chen T, Huang QY, Weir BA, Sizemore A (2019). Small-molecule targeting of brachyury transcription factor addiction in chordoma. Nat Med.

[15] Sheppard HE, Dall’Agnese A, Park WD, Shamim MH, Dubrulle J, Johnson HL (2021). Targeted brachyury degradation disrupts a highly specific autoregulatory program controlling chordoma cell identity. Cell Rep Med.

[16] Alexandrov LB, Nik-Zainal S, Wedge DC, Aparicio SA, Behjati S, Biankin AV (2013). Signatures of mutational processes in human cancer. Nature.

[17] Douville C, Springer S, Kinde I, Cohen JD, Hruban RH, Lennon AM (2018). Detection of aneuploidy in patients with cancer through amplification of long interspersed nucleotide elements (LINEs). Proc Natl Acad Sci U S A.

[18] Hu Y, Mintz A, Shah SR, Quinones-Hinojosa A, Hsu W (2014). The FGFR/MEK/ERK/brachyury pathway is critical for chordoma cell growth and survival. Carcinogenesis.

[19] Wilbie D, Walther J, Mastrobattista E (2019). Delivery aspects of CRISPR/Cas for in vivo genome editing. Acc Chem Res.

[20] Schumann K, Lin S, Boyer E, Simeonov DR, Subramaniam M, Gate RE (2015). Generation of knock-in primary human T cells using Cas9 ribonucleoproteins. Proc Natl Acad Sci U S A.

[21] Fajrial AK, He QQ, Wirusanti NI, Slansky JE, Ding X (2020). A review of emerging physical transfection methods for CRISPR/Cas9-mediated gene editing. Theranostics.

[22] Lyu P, Javidi-Parsijani P, Atala A, Lu B (2019). Delivering Cas9/sgRNA ribonucleoprotein (RNP) by lentiviral capsid-based bionanoparticles for efficient ‘hit-and-run’ genome editing. Nucleic Acids Res.

[23] Lu Z, Yao X, Lyu P, Yadav M, Yoo K, Atala A (2021). Lentiviral capsid-mediated Streptococcus pyogenes Cas9 ribonucleoprotein delivery for efficient and safe multiplex genome editing. CRISPR J..

[24] Strober W (2015). Trypan Blue Exclusion Test of cell viability. Curr Protoc Immunol.

[25] Zhou J, Wang J, Shen B, Chen L, Su Y, Yang J (2014). Dual sgRNAs facilitate CRISPR/Cas9-mediated mouse genome targeting. FEBS J.

[26] Ingham MHJ, Whalen GF, Thomas JS, El-Khoueiry AB, Hanna DL, Olszanski AJ, Meyer CF, Azad NS, Mahmood S et al. Early results of intratumoral INT230-6 alone or in combination with ipilimumab in subjects with advanced sarcomas. 2021 ASCO Annual Meeting. McCormick Place, Chicago, Illinois. 2021. p. 1.

[27] Rasul MF, Hussen BM, Salihi A, Ismael BS, Jalal PJ, Zanichelli A (2022). Strategies to overcome the main challenges of the use of CRISPR/Cas9 as a replacement for cancer therapy. Mol Cancer.

[28] Wei T, Cheng Q, Min YL, Olson EN, Siegwart DJ (2020). Systemic nanoparticle delivery of CRISPR-Cas9 ribonucleoproteins for effective tissue specific genome editing. Nat Commun.

[29] Duan L, Ouyang K, Xu X, Xu L, Wen C, Zhou X (2021). Nanoparticle delivery of CRISPR/Cas9 for genome editing. Front Genet.

[30] Glass Z, Lee M, Li Y, Xu Q (2018). Engineering the Delivery System for CRISPR-Based genome editing. Trends Biotechnol.

[31] Hamilton JR, Tsuchida CA, Nguyen DN, Shy BR, McGarrigle ER, Sandoval Espinoza CR (2021). Targeted delivery of CRISPR-Cas9 and transgenes enables complex immune cell engineering. Cell Rep.

[32] Mangeot PE, Risson V, Fusil F, Marnef A, Laurent E, Blin J (2019). Genome editing in primary cells and in vivo using viral-derived Nanoblades loaded with Cas9-sgRNA ribonucleoproteins. Nat Commun.

[33] Banskota S, Raguram A, Suh S, Du SW, Davis JR, Choi EH (2022). Engineered virus-like particles for efficient in vivo delivery of therapeutic proteins. Cell.

[34] Park H, Oh J, Shim G, Cho B, Chang Y, Kim S (2019). In vivo neuronal gene editing via CRISPR-Cas9 amphiphilic nanocomplexes alleviates deficits in mouse models of Alzheimer’s disease. Nat Neurosci.

[35] Ekman FK, Ojala DS, Adil MM, Lopez PA, Schaffer DV, Gaj T (2019). CRISPR-Cas9-Mediated genome editing increases Lifespan and Improves Motor deficits in a Huntington’s Disease Mouse Model. Mol Ther Nucleic Acids.

[36] Yin D, Ling S, Wang D, Dai Y, Jiang H, Zhou X (2021). Targeting herpes simplex virus with CRISPR-Cas9 cures herpetic stromal keratitis in mice. Nat Biotechnol.

[37] Geisinger JM, Stearns T (2020). CRISPR/Cas9 treatment causes extended TP53-dependent cell cycle arrest in human cells. Nucleic Acids Res.

[38] Yin H, Kauffman KJ, Anderson DG (2017). Delivery technologies for genome editing. Nat Rev Drug Discov.

[39] Liu C, Zhang L, Liu H, Cheng K (2017). Delivery strategies of the CRISPR-Cas9 gene-editing system for therapeutic applications. J Control Release.

[40] WHO (2020). Classification of soft tissue and bone tumours.

[41] Karele EN, Paze AN, Chordoma (2022). To know means to recognize. Biochim Biophys Acta Rev Cancer.

[42] Hung YP, Diaz-Perez JA, Cote GM, Wejde J, Schwab JH, Nardi V (2020). Dedifferentiated Chordoma: clinicopathologic and molecular characteristics with integrative analysis. Am J Surg Pathol.

[43] Trucco MM, Awad O, Wilky BA, Goldstein SD, Huang R, Walker RL (2013). A novel chordoma xenograft allows in vivo drug testing and reveals the importance of NF-kappaB signaling in chordoma biology. PLoS ONE.

[44] Shih AR, Cote GM, Chebib I, Choy E, DeLaney T, Deshpande V (2018). Clinicopathologic characteristics of poorly differentiated chordoma. Mod Pathol.

[45] Rekhi B, Michal M, Ergen FB, Roy P, Puls F, Haugland HK (2021). Poorly differentiated chordoma showing loss of SMARCB1/INI1: clinicopathological and radiological spectrum of nine cases, including uncommon features of a relatively under-recognized entity. Ann Diagn Pathol.

[46] Makise N, Shimoi T, Sunami K, Aoyagi Y, Kobayashi H, Tanaka S (2022). Loss of H3K27 trimethylation in a distinct group of de-differentiated chordoma of the skull base. Histopathology..

