# Supplementary material for: Virus-like particle-based delivery of Cas9/guide RNA ribonucleoprotein efficiently edits the brachyury gene and inhibits chordoma growth in vivo
Source: Discov Oncol. 2023 May 18;14:70. doi: 10.1007/s12672-023-00680-9 (PMC10192467; doi:10.1007/s12672-023-00680-9)
Supplement: Supplementary file 1 — Additional file 1 [file 12672_2023_680_MOESM1_ESM.docx]

**Title**

Virus-like particle-based delivery of Cas9/guide RNA ribonucleoprotein efficiently edits the brachyury gene and inhibits chordoma growth *in vivo*

**Authors**

Yunping Hu^1^*, Baisong Lu^2^, Zhiyong Deng^3^, Fei Xing^4^, Wesley Hsu^1^*

**Affiliations**

^1^ Department of Neurological Surgery, Wake Forest University School of Medicine, Medical Center Boulevard, Winston-Salem, NC 27157, USA

^2^ Wake Forest University Institute for Regenerative Medicine, Medical Center Boulevard, Winston-Salem, NC 27157, USA

^3^ Department of Physiology and Pharmacology, Wake Forest University School of Medicine, Medical Center Boulevard, Winston-Salem, NC 27157, USA

^4^ Department of Cancer Biology, Wake Forest University School of Medicine, Medical Center Boulevard, Winston-Salem, NC 27157, USA

***Corresponding authors**

Wesley Hsu, email: [whsu@wakehealth.edu](mailto:whsu@wakehealth.edu)

Yunping Hu, email:yhu@wakehealth.edu

**Supplementary Data**

**Supplementary Table S1. Sequences of sgRNA targeting brachyury**

| **Gene name** |  | **Primers** |
| --- | --- | --- |
| sgRNA a | forward | 5’-ACCGggcctacctggtcagtgtcg-3’ |
|  | reverse | 5’-AAACcgacactgaccaggtaggcc-3’ |
| sgRNA b | forward | 5′- ACCGgtgctgaaggtgaacgtgtc-3’ |
|  | reverse | 5’- AAACgacacgttcaccttcagcac-3’ |
| sgRNA c | forward | 5’-CACCGtagcgcgtctccccgctcctc-3’ |
|  | reverse | 5’-AAACgaggagcggggagacgcgctaC-3’ |
| sgRNA d | forward | 5’-CACCgcaggaagccttcctctcagt-3’ |
|  | reverse | 5’-AAACactgagaggaaggcttcctgc-3’ |

**Supplementary Table S2. Primers for quantitative real-time PCR**

| **Gene name** |  | **Primers** |
| --- | --- | --- |
| Brachyury | forward | 5’-AGACTGGAGAGTTGGG-3’ |
|  | reverse | 5’- CAGGTGGTCCACTCGGTACT-3’ |
| Cas9 | forward | 5’-GGAACCGCTGGAGAGCAACT-3’ |
|  | reverse | 5’-GTCCCTATCGAAGGACTCTGGCA-3’ |
| GAPDH | forward | 5′-CATGAGAAGTATGACAACAGCCT-3′ |
|  | reverse | 3′-AGTCCTTCCACGATACCAAAGT-5′ |

**Supplementary Fig. 1S**

**Brachyury gRNA**

**Marker Vehicle a+c a+d b+c b+d**


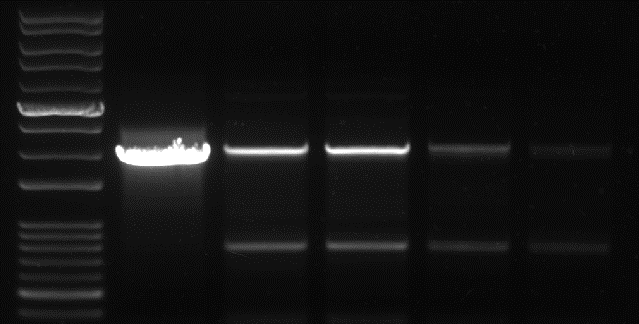


**1k**

**800**

**700**

**3k**

**2k**

**Wild type/Brachyury**

**Deletion**

The representative PCR analysis showing deletion caused by brachyury sgRNAs.

**Supplementary Fig. 2**

**JHC7**

**UCH2**

**Time (h) 0 12 24 48 96**

**Time (h) 0 12 24 48 96**


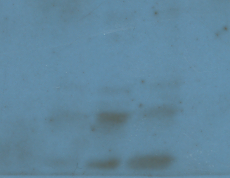

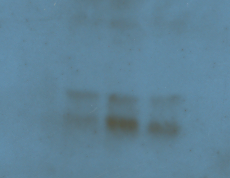


**160 kDa**

**45 kDa**

**160 kDa**

**Cas9**

**β-actin**

**Cas9**


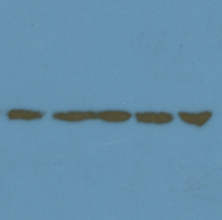

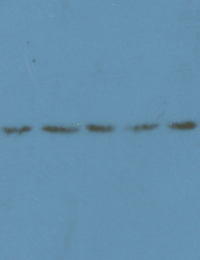


**45 kDa**

**β-actin**

The representative Western blot analysis of Cas9 expression in JHC7 and UCH2 cells treated with VLP-packaged brachyury gRNA a+c (50 ng) for up to 96 h.

**a**

**Supplemental Fig. S3**

**JHC7**

**VLP-packaged brachyury gRNA**

**Marker Vehicle a+c a+d b+c b+d**


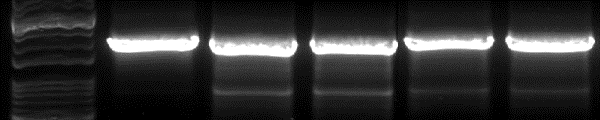


**3k**

**2k**

**Wild type/Brachyury**

**1k**

**Deletion**

**800**

**700**

**UCH2**

**VLP-packaged brachyury gRNA**

**Marker Vehicle a+c a+d b+c b+d**


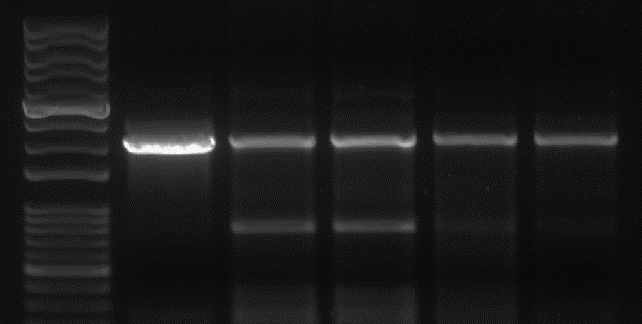


**3k**

**Wild type/Brachyury**

**2k**

**1k**

**Deletion**

**700**

**800**

**VLP-packaged brachyury gRNA**

**UCH2**

**JHC7**

**b**

**VLP-packaged brachyury gRNA**

**Vehicle a+c a+d b+c b+d**

**Vehicle a+c a+d b+c b+d**


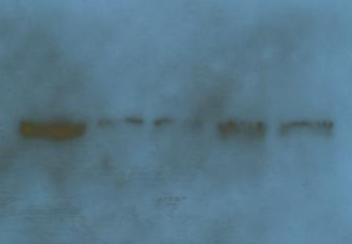

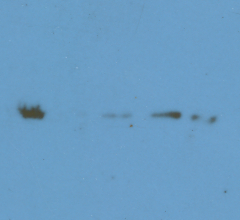


**Brachyury**


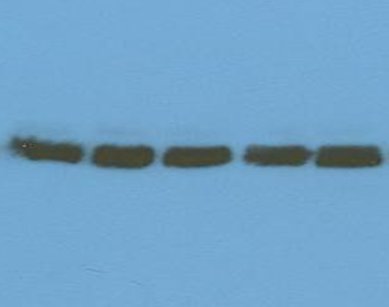

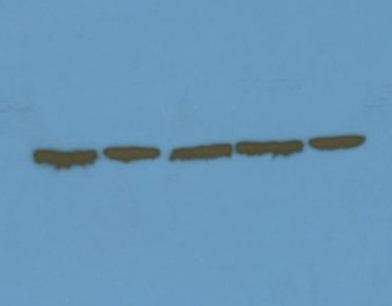


**β-actin**


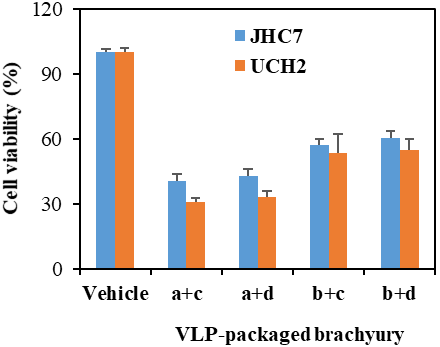


#

#

#

#

*

*

*

*

**c**

**a.** The representative PCR analysis showing deletion caused by VLP-packaged brachyury gRNAs in JHC7 and UCH2 cells. **b.** The representative Western blot analysis of brachyury expression in JHC7 and UCH2 cells treated with vehicle (medium) or VLP-packaged brachyury gRNAs (50 ng) for 48 h. **c.** JHC7 and UCH2 cells were treated with VLP-packaged brachyury gRNAs (50 ng) for 48 h. Cell viability was measured by trypan blue exclusion assay. Data were presented as mean ± SD (n=3). * P < 0.05, vs Vehicle in the same cell line; # P < 0.05, vs a+c or a+d in the same cell line.
